# Proteome-wide search for functional motifs altered in tumors: Prediction of nuclear export signals inactivated by cancer-related mutations

**DOI:** 10.1038/srep25869

**Published:** 2016-05-12

**Authors:** Gorka Prieto, Asier Fullaondo, Jose A. Rodríguez

**Affiliations:** 1 University of the Basque Country (UPV/EHU), Department of Communications Engineering, Bilbao, 48013, Spain; 2 University of the Basque Country (UPV/EHU), Department of Genetics, Physical Anthropology and Animal Physiology, Leioa, 48940, Spain

## Abstract

Large-scale sequencing projects are uncovering a growing number of missense mutations in human tumors. Understanding the phenotypic consequences of these alterations represents a formidable challenge. *In silico* prediction of functionally relevant amino acid motifs disrupted by cancer mutations could provide insight into the potential impact of a mutation, and guide functional tests. We have previously described Wregex, a tool for the identification of potential functional motifs, such as nuclear export signals (NESs), in proteins. Here, we present an improved version that allows motif prediction to be combined with data from large repositories, such as the Catalogue of Somatic Mutations in Cancer (COSMIC), and to be applied to a whole proteome scale. As an example, we have searched the human proteome for candidate NES motifs that could be altered by cancer-related mutations included in the COSMIC database. A subset of the candidate NESs identified was experimentally tested using an *in vivo* nuclear export assay. A significant proportion of the selected motifs exhibited nuclear export activity, which was abrogated by the COSMIC mutations. In addition, our search identified a cancer mutation that inactivates the NES of the human deubiquitinase USP21, and leads to the aberrant accumulation of this protein in the nucleus.

Global sequencing projects, such as The Cancer Genome Atlas, are uncovering a large number of cancer-related genomic alterations, including many missense mutations that result in single amino acid changes at the protein level. Some of these mutations may affect previously studied and well-characterized protein domains, such as the p53 DNA binding domain^1^, but, in most cases, the potential functional consequences of missense mutations are not immediately apparent.

A number of *in silico* approaches based on evolutionary sequence conservation or structural constraints, such as SIFT^2^ or Polyphen-2^3^ can provide general insight into the potential deleterious effects of a mutation, although the accuracy of some of these methods has been recently reported to be limited^4^.

A variety of computational tools, such as ELM^5^, have been developed to predict potential functional motifs in proteins. These tools, when combined with the data available in repositories such as the Cataloque of Somatic Mutations in Cancer (COSMIC)^6^ may hint to the functional or regulatory aspects of a protein that could be disrupted by cancer-related mutations, and therefore be useful to guide subsequent functional tests. When applied at a global genome or proteome scale, functional motif prediction combined with cancer mutation database search may contribute to understand the phenotypic consequences of the complex molecular landscape that characterizes human tumors.

Nuclear localization signals (NLSs) and nuclear export signals (NESs) represent two prominent examples of amino acid sequence motifs with important functional and regulatory implications. These motifs are recognized and bound by karyopherin family receptors (importins or exportins) that mediate the shuttling of proteins between the nucleus and cytoplasm^7^. Nucleocytoplasmic transport of proteins is an essential process for cell homeostasis, and can be dysregulated in cancer^8^. In particular, mutations in NLSs or NESs leading to aberrant subcellular distribution of the cognate protein have been identified in tumor cells^9^,^10^. Therefore, nucleocytoplasmic transport is currently emerging as a promising therapeutic target in cancer^11^,^12^.

The best characterized and seemingly most abundant type of nuclear export signal is the so-called leucine-rich NES (hereafter referred to as NES, for simplicity), which is recognized by the exportin CRM1. This NES/CRM1 complex mediates the nuclear export of many proteins, including the products of several prominent tumor suppressors and oncogenes^13^. NESs are formed by a series of hydrophobic amino acids with a characteristic spacing that loosely fits the consensus pattern 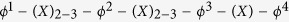, where *ϕ* represents a hydrophobic residue and X represents any residue. *In silico* identification of potential of NESs is remarkably challenging, and several computational tools have been developed over the last years to assist in NESs prediction^14^,^15^,^16^,^17^. We have previously reported Wregex 1.0 (http://ehubio.ehu.eus/wregex), which combines a regular expression with a Position-Specific Scoring Matrix (PSSM), as a tool for candidate NES prediction that can be also applied to prediction of other linear motifs^16^. In addition to its flexibility, a prominent feature of Wregex is its fast execution time, which allows its combination with large databases, and its application to global proteome data.

Here we present a series of improvements to Wregex to enable its combination with the COSMIC mutation database and its use in large scale screenings of potential functional motifs targeted by cancer mutations. As a proof-of-concept, we have carried out a proteome-wide prediction of novel candidate NES motifs that could be inactivated by cancer-related mutations. A subset of the identified motifs was selected for subsequent experimental testing. When tested using an *in vivo* nuclear export assay^18^, a significant proportion of the selected motifs exhibited nuclear export activity, which was abrogated by cancer-related mutations. Finally, we identify a cancer mutation (L144H) that inactivates the NES of the human deubiquitinase USP21^19^, and causes its aberrant accumulation in the nucleus.

## Results and Discussion

### Wregex improvements

**Figure 1:**
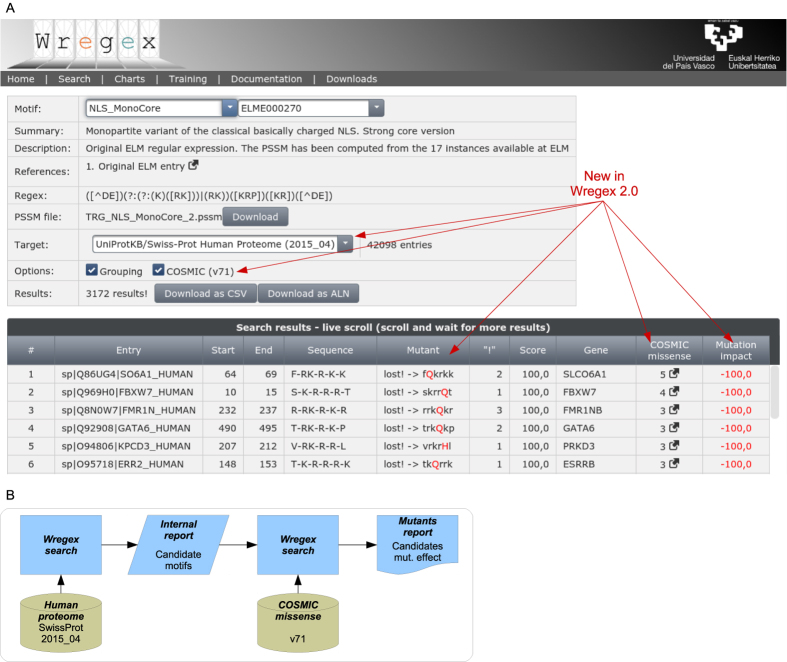
Improved Wregex web interface for large scale mutants screening. (**A**) Screenshot of the Wregex 2.0 search tab (UPV/EHU logo courtesy of the University of the Basque Country). After selecting the desired motif, a large scale screening is configured by targeting the whole human proteome from UniProt/SwissProt. Mutant analysis is configured by clicking the COSMIC option. When this option is enabled, three new columns are displayed: *Mutant*, with the mutated sequence; *COSMIC missense*, with the number of recurrent missense mutations found in COSMIC; *Mutation impact*, with the effect of the mutation in the predicted candidate score. The candidate list is ranked according to both mutation impact and number of recurrent mutations. (**B**) Workflow of search for candidate functional motifs with COSMIC mutation. Two Wregex searches are combined. Firstly, a FASTA database with human protein sequences is used as target, and a internal candidate list is obtained. Secondly, mutations from COSMIC are applied to the internal candidate list, which is used as the new target. Candidate motifs with their mutation impact are finally reported.

As shown in Fig. 1A, the Wregex 2.0 web interface has been updated to include mutation information from the COSMIC database^6^. When [COSMIC] is enabled as option, two Wregex searches are combined (Fig. 1B). A first search for candidate functional motifs is carried out against the target fasta. Then, COSMIC is checked for missense mutations in each of the obtained results. If mutations are found, a second Wregex search is performed for each mutant and the result is replaced by a list of results including the effect of each mutation in Wregex score. These results include also the number of tumor samples bearing the mutation, as described in COSMIC, and a link to the corresponding COSMIC page. The final candidate list is ranked according to the impact and the prevalence of the mutation (recurrent mutations with the highest impact are ranked fist). These results can be downloaded as a CSV file and filtered/reordered using any spreadsheet program. It must be noted that this approach can be helpful to predict mutational disruption of existing functional motifs, but it will not allow identifying alterations that result in the appearance of new motifs, such as the frameshift mutations that create novel NESs in nucleophosmin^20^.

**Figure 2:**
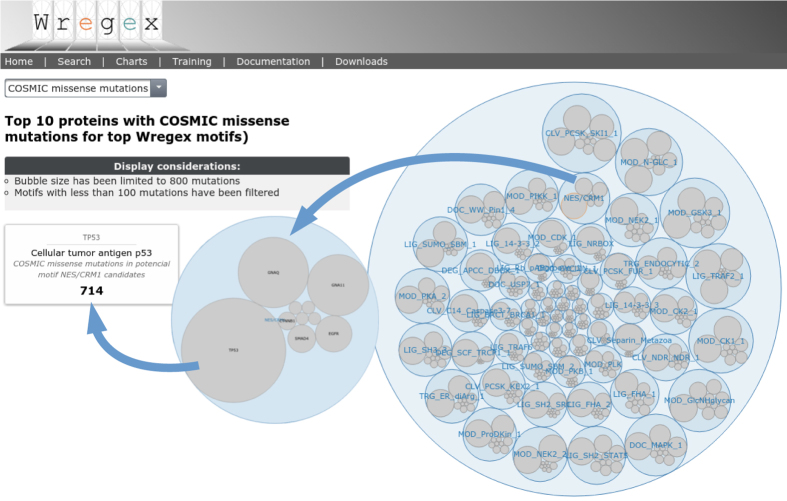
Wregex chart view of recurrent COSMIC mutations for predicted candidates using all ELM motifs using as “Target” the human proteome. A Wregex search is carried out for each motif available at ELM, targeting the whole human proteome from UniProt/SwissProt. Recurrent mutations found in COSMIC for the predicted candidate motifs are displayed. Each blue bubble represents an ELM motif, and the gray bubbles inside represent the genes with a larger number of recurrent missense mutations in their candidate motifs positions. The bubble size is proportional to the number of mutations (until 800 for displaying purposes). If the user clicks in any bubble, it is zoomed in and the corresponding information is displayed.

The high processing speed of Wregex, makes it feasible to search the whole human proteome for the potential effect of COSMIC database mutations on every linear motif available at ELM^5^. Wregex displays the result of this search in an easy-to-browse bubble chart in the “Charts” tab (Fig. 2). In the chart, each different ELM motif is represented by a bubble, which contains several other bubbles, each representing a gene bearing mutations in the corresponding motif. The size of the bubble is proportional to the number of mutations described in COSMIC. If the user clicks in any bubble, it is zoomed in and information is displayed.

### Using Wregex to predict candidate NESs and NLSs altered by cancer mutations

**Figure 3:**
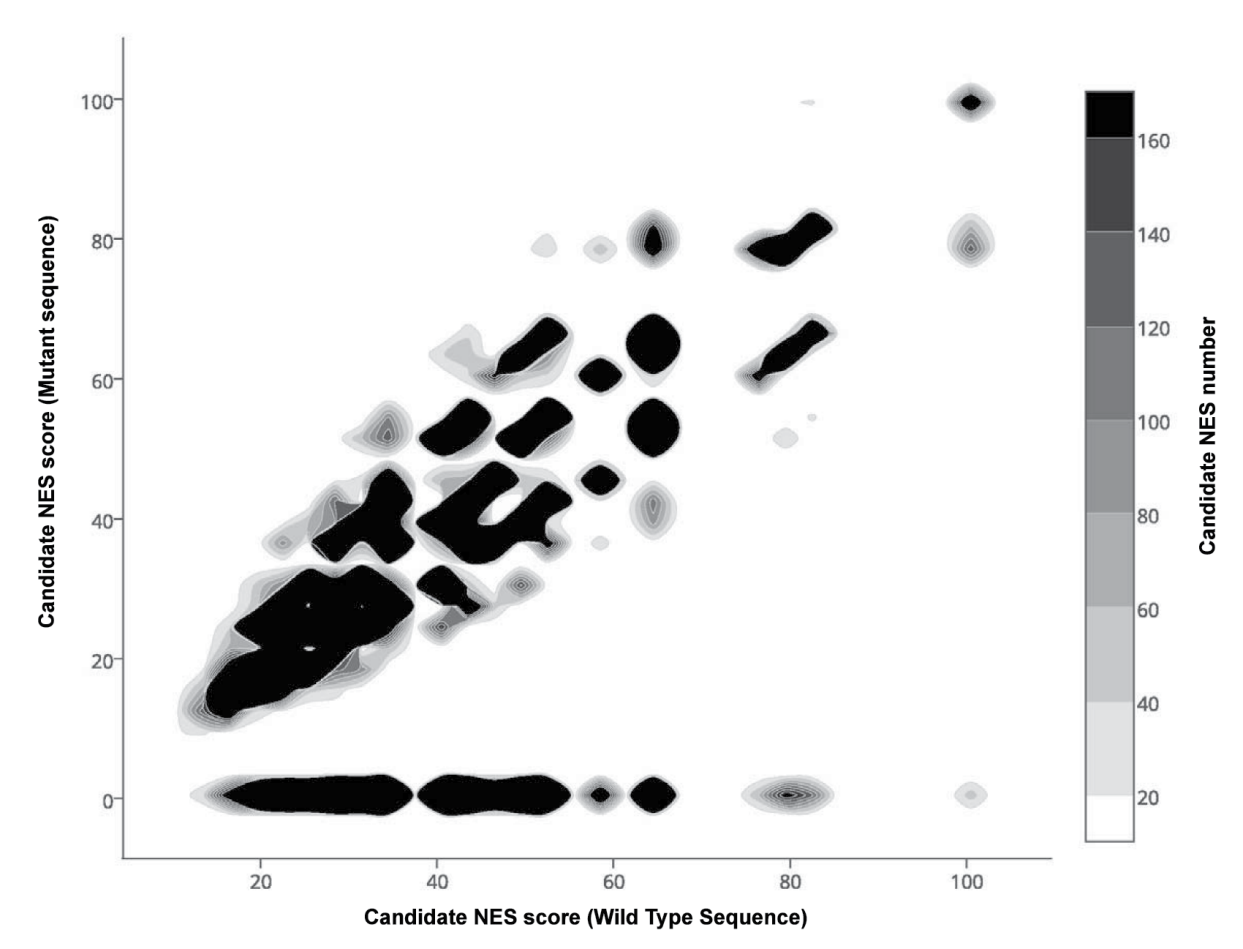
Mutation impact of predicted NES/CRM1 motifs for the whole human proteome. Wregex was used to search the whole human proteome from UniProt/SwissProt (release 2015_04), using the relaxed configuration for NES/CRM1 motif, and enabling the COSMIC (release v71) mutations support. This search resulted into 214988 COSMIC missense mutations altering potential NES/CRM1 motifs. In the x-axis the Wregex score of the wild type candidate sequence is displayed. The y-axis displays the Wregex score of the candidate sequence after substituting an amino acid with a COSMIC missense mutation. Points near the diagonal line indicate no significative difference between the mutant and wild type sequences predicted scores. Points above the line indicate mutants with NES activity gain, and the points below are mutants with NES activity loss. In this case, the most interesting candidates are those in the lower-right part of the graph, since their wild type sequence has a high score and the mutant is predicted to completely loss the function.

To illustrate Wregex capabilities, we present the results of proteome-wide search for potential NESs that might be affected by COSMIC mutations. On the Wregex web interface, the [NES/CRM1] was selected as “Motif” and the [UniProt/SwissProt Human Proteome (2015_04)] as “Target”. The [COSMIC] option was also selected. In this first step we use the relaxed configuration, as we noted that mutations in non-significant positions could be erroneously interpreted as resulting in loss-of-function when using a very strict regular expression for motif definition. Using this approach, a total of 214988 candidates were obtained. Figure 3 displays the predicted NES activity score for the wild type (x-axis) and the mutant (y-axis) variants of each candidate. The lower right area of the graph includes those candidates whose NES activity score is most drastically reduced by the mutation, and would therefore be of highest interest for subsequent follow up studies.

As a further example of a proteome-wide analysis using Wregex, a similar screening was carried out to search for potential NLSs that might be targeted by cancer mutations. The details and the results of this analysis are presented in Additional File 1 and Additional File 2.

These datasets constitute a starting point to guide further research on the role of the nucleocytoplasmic transport in cancer.

### Selecting candidate NESs for experimental testing

**Figure 4:**
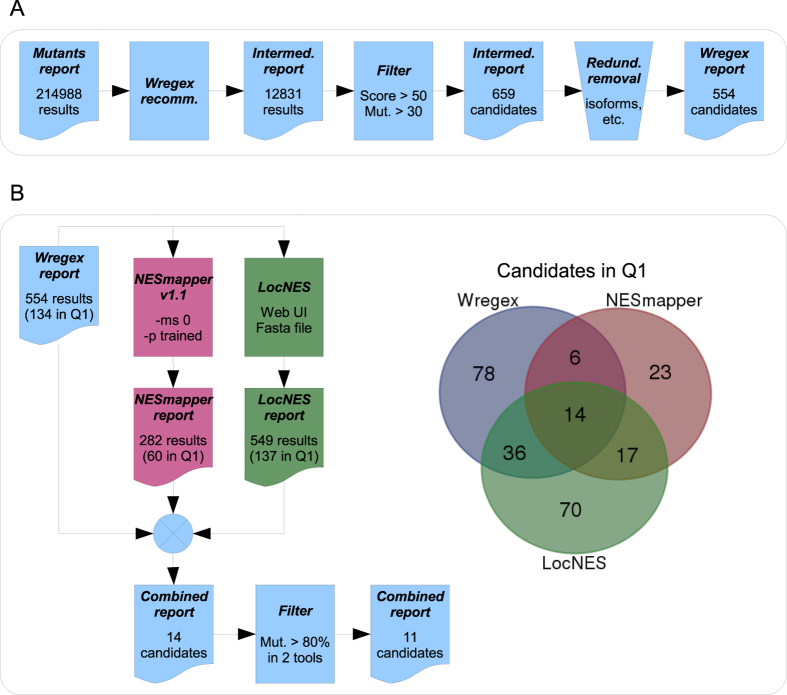
Selection of a subset candidate NES motifs for experimental validation. (**A**) Workflow for selecting candidate NES motifs using Wregex. A new Wregex search was carried out targeting the original candidates list but using the recommended (strict) configuration for the NES/CRM1 motif in order to decrease the number of false positives. This resulted into a 12831 candidate lists, which was then narrowed by selecting only those candidates with a wild type score greater than 50 and a mutation impact greater than 30. Finally redundancies due to isoforms were removed resulting into a 554 candidates list. (**B**) Workflow for selecting a subset candidate list for experimental testing. The narrowed Wregex candidates list was processed using two new NES prediction tools: NESmapper and LocNES, resulting into 282 and 549 candidates respectively. The best candidates predicted by the three tools were selected by ranking then according to their score and considering only those in the first quartile (Q1). As illustrated by the Venn diagram, there were only 14 candidates in Q1 in all of the three tools, which were reduced to 11 after requiring a mutation impact greater than 80% in two of the tools.

In order to select a subset of candidate NES targeted by cancer mutations for experimental testing, a series of filters were applied to the large initial candidate list in two separate steps (Fig. 4). First (Fig. 4A), the candidate list was narrowed using the recommended Wregex configuration because of its specificity. In this recommended configuration a strict regular expression is used, which significantly decreases the number of candidates, but minimizes the number of false positives. Those candidates with a Wregex score greater than 50 and a mutation effect greater than 30 were selected, and isoform redundancies were removed. This first step of filtering produced a list of 554 candidates in 532 genes, called “Wregex candidate list” (Additional File 3). Of note, one of the highest-ranking candidates in this list is a previously described NES in the human deubiquitinase USP21^19^. Our Wregex analysis predicts that a mutation identified in a large intestine carcinoma (L144H) abrogates the activity of USP21 NES.

With this list we provide a dataset of preselected candidate NESs with missense mutations described in COSMIC that are predicted to have an impact on their NES activity.

A second filtering step (Fig. 4B) was next used to select a smaller number of *a priori* optimal candidates, feasible for experimental testing. In a previous comparative analysis^19^, we noted that combining the results of different NES prediction programs might be a useful strategy to select NES candidates more likely to be functional. Taking this into account, we decided to examine the proteins included in the “Wregex candidate list” using other NES prediction tools. As a preliminary test, we compared the performance of the combination of Wregex with the three most recent NES prediction tools: NESmapper^15^, LocNES^14^ and NESsential^17^ using ValidNESs^21^, a well-curated database of validated CRM1-dependent NESs, as a test dataset. As shown in Additional File 4, this analysis indicated that the combination with LocNES provided the highest number of true positives, although the true positive rate (TPR) was similar among the different combinations. On the other hand, the combination with NESsential resulted in the highest false positive rate (FPR), while the lowest FPR was obtained by using the combination with NESmapper. Thus, we concluded that the combination of Wregex with NESsential provides a similar sensitivity, but a lower specificity than the combination with the other two tools. Therefore, the final analysis was carried out using the combination of Wregex with NESmapper and LocNES. The complete information is provided as Additional File 5. As shown in Fig. 4B, NESmapper identified 282 candidate NES (60 in the first quartile, Q1) and LocNES identified 549 candidates (137 in Q1) in this set of proteins. 14 common candidates that rank highest (Q1) in the prediction by the three programs were identified. For 11 of these candidates, the effect of the mutation was greater than 80% (predicting likely mutational inactivation of the NES) according to at least two of the programs. These 11 candidates, along with the mutant version of the previously reported USP21 NES were selected for testing (Table 1).

### Experimental validation of predicted NESs inactivated by cancer mutations

**Figure 5:**
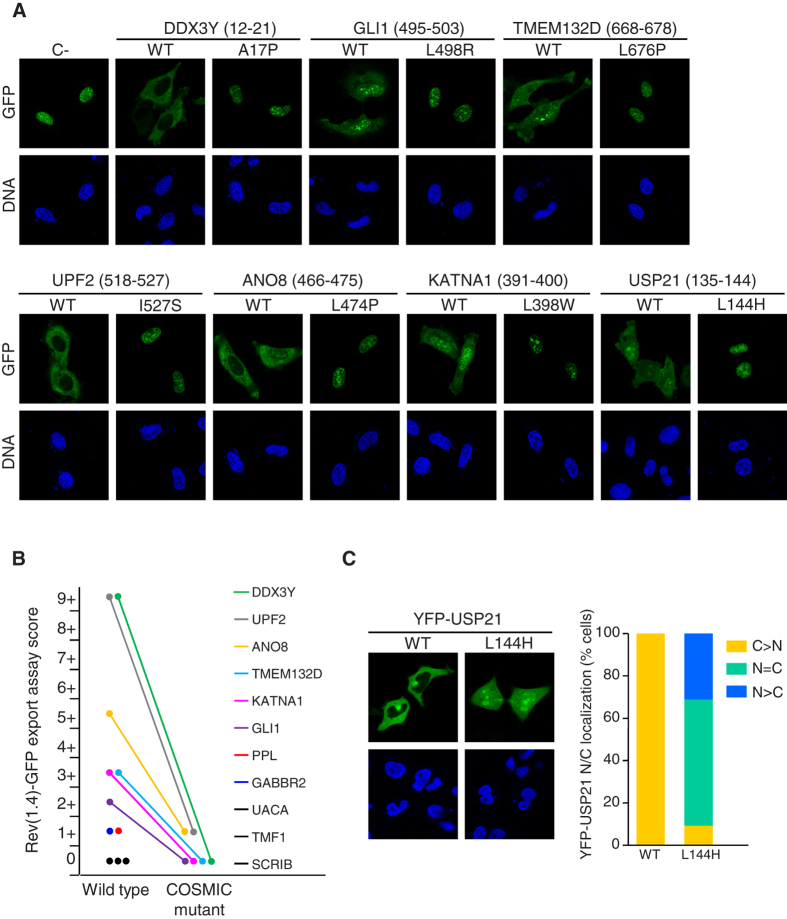
Experimental validation of *in silico* predicted NESs-inactivating cancer mutations. (**A**) Images show the results of an *in vivo* nuclear export assay to test the activity of the wild type (WT) and mutant versions of NES sequences predicted in the indicated proteins. The position of the predicted NES is indicated between brackets. HeLa cells were transfected with pRev(1.4)–GFP-based plasmids encoding WT and mutant version of each candidate NES. The empty pRev(1.4)–GFP plasmid was included as a negative control (C–). GFP panels show representative examples of the localization of the GFP-tagged proteins and DNA panels show the cell nuclei stained with DAPI. Active NES motifs induce a partial or complete relocation of the GFP signal to the cytoplasm, which is abrogated by cancer-related mutations. (**B**) Graph summarizing the results of the nuclear export assay. The export assay score for the wild type and the COSMIC mutant version of each candidate NES was calculated as described in^18^. The activity of the mutant version was only evaluated for those NESs whose WT version had an export assay score ≥2+. In all cases, the mutation either completely abrogated or drastically reduced the export activity. (**C**) Effect of a cancer-related NES mutation (L144H) on the nucleocytoplasmic (NC) localization of full length USP21. Images on the left show representative examples of HeLa cells expressing YFP-USP21WT or YFP-USP21L144H. Graph shows the percentage of cells having predominantly cytoplasmic (C > N), predominantly nuclear (N > C) or equally nuclear and cytoplasmic (N = C) localization of YFP-USP21. 200 cells were counted per sample.

An *in vivo* nuclear export assay^18^ was used to evaluate the activity of the candidate NES shown in Table 1. As shown in Fig. 5A,B, 6 out of the 11 candidates [DDX3Y (12–21), GLI1 (495–503), TMEM132D (668–678), UPF2 (518–527), ANO8 (466–475) and KATNA1 (391–400)] showed an export assay activity score ≥2+. In line with the *in silico* prediction, the export activity of these signals was fully abrogated or drastically reduced by the cancer mutation. Two other candidate NESs [PPL (967–976) and GABBR2 (805–814)] showed a borderline (1+) export activity and three candidates [UACA (529–538) TMF1 (714–723) and SCRIB (262–270)] tested negative in the assay. In these five cases the effect of the mutation was not evaluated. These experimental results show a reasonably good agreement with the *in silico* predictions, thus supporting the validity of our computational approach.

In this study, we have focused on the methodology to predict putative functional motifs targeted by cancer mutations. It remains to be experimentally evaluated to what extent the novel candidate NES motifs reported here contribute to determine the localization of their cognate proteins. A first step to confirm their relevance would be to introduce these mutations in the full-length protein. However, it must be taken into account that nucleocytoplasmic transport is a complex cellular process, subject to multiple levels of control^22^. For example, in the context of the full-length protein, the exposure and activity of these motifs could be regulated by masking or by post-translational modification, such as phosphorylation, as previously described for other NESs^23^,^24^. Therefore, a comprehensive set of experiments will be probably necessary to precisely determine the physiological role of the identified candidate NESs, as well as the pathological consequences of the cancer mutations in these sequences.

In the case of full-length USP21, we have previously shown that its cytoplasmic localization results from CRM1-dependent nuclear export mediated by the NES [USP21 (135–144)], and that experimental mutations in this sequence abrogate USP21 shuttling^19^. We therefore set out to determine the effect that the cancer mutation L144H, predicted *in silico* to disrupt this NES, might have on the regulation of USP21 nucleocytoplasmic distribution. First, as illustrated in Fig. 5A, we used the nuclear export assay to confirm that this mutation does indeed abrogate the export activity of USP21 NES, previously reported to be 4+ in this assay^19^. Next, we used site directed mutagenesis to introduce the L144H mutation into full-length YFP-USP21. As shown in Fig. 5C, this mutation led to a marked increase in the nuclear accumulation of YFP-USP21.

## Conclusion

In this study we present a novel approach to search the human proteome for functional motifs mutationally altered in tumors. This approach exploits the capability of Wregex to be combined with other databases and to process large datasets. Using the NES/CRM1 interacting motif as a proof-of-concept, we have searched the whole human proteome for potential candidate NESs that may be targeted by cancer-related mutations, and we have carried a limited experimental validation of the predictions. We identify six novel putative NESs inactivated in cancer, and we show that a cancer mutation predicted to disrupt the NES of USP21 leads to the aberrant nuclear accumulation of this protein. These findings illustrate the potential of our approach to identify cancer mutations that may alter protein function or regulation.

## Methods

### Combining Wregex with COSMIC mutations

COSMIC information was obtained by using the “CosmicMutantExport” TSV file available from the “Data Download” tab of the COSMIC web site. We considered only missense mutations, so those entries with no value in the “Mutation AA” field were discarded. We then processed this field using the Human Genome Variation Society recommendations^25^ for the description of protein sequence variants (v2.0), and considered only single amino acid missense mutations. Entries referring the same gene, the same position and wild type amino acid, and the same mutated amino acid were considered as recurrent mutations.

### Using Wregex to search the human proteome for candidate NES targeted by cancer mutations

Wregex was used to search the whole human proteome from UniProt/SwissProt (release 2015_04) for “NES/CRM1” motifs using the relaxed configuration. Missense mutations that alter any residue of a candidate NES, were retrieved from COSMIC (release v71) and Wregex was applied to the mutant amino acid sequence. The output was a list of 214988 results, each consisting of paired wild type and mutant versions of a candidate NES sequence, including the score of the wild type sequence (maximum is 100) and the effect of the mutation (e.g. -100 if the wild type motif had the maximum score and its function is predicted to be lost in the mutant sequence). In this first step, the recommended NES/CRM1 configuration, which applies a more strict regular expression, was not used to prevent mutations in non-critical positions being reported as loss-of-function.

This large initial list was subsequently filtered, by applying the recommended (strict) Wregex configuration to wild type sequences, which resulted in a shorter list of 12831 results. Next, we selected those candidates fulfilling the following two criteria: (1) the Wregex (strict) score of the wild type sequence is higher than 50, and (2) the Wregex (relaxed) score of the COSMIC mutant decreases at least in 30. This filtering produced a new list of 659 candidates. After manually removing redundancies (due to isoforms), a final “Wregex candidate list” of 554 motifs ranked according to the impact of the mutation on the Wregex score was obtained (Additional File 3).

### Selection of candidate NESs for experimental testing

On one hand, by visual inspection of the Wregex candidate list, we noted the presence of a previously described functional NES that mediates nuclear export of the USP21 deubiquitinase^19^. This candidate was ranked 16 (mutation impact of −100), and was selected for assay. On the other hand, the proteins bearing the remaining 553 Wregex candidates were examined using both NESmapper^15^ (using minscore of 0 and trained profile) and LocNES^14^ NES prediction tools.

To select the fraction of candidates in the first quartile (Q1), we sorted the scores of the candidates and calculated a threshold score containing the 25% higher-score candidates. This was accomplished using the Excel QUARTILE() function using “3” as the second parameter. Then, we selected the candidates with a score higher than that threshold. It must be noted that, in the case of Wregex, the threshold score was shared by several candidates. Thus, the number of Q1 candidates for Wregex represents 24.18% of the total number.

### Experimental procedures

Candidate NESs were tested using a previously described *in vivo* nuclear export assay^18^. To this end, double stranded DNA fragments encoding the wild type and mutant versions of the candidate NES were cloned into the pRev(1.4)–GFP vector (a gift from Dr. Beric Henderson). HeLa cells, growing in Dulbecco’s modified Eagle’s medium, supplemented with 10% fetal bovine serum, 100 units/ml penicillin and 100 *μ*g/ml streptomycin (all from Invitrogen), were seeded onto sterile glass coverslips in 12-well trays. Cells were transfected with the pRev(1.4)–GFP-based plasmids containing the candidate NESs using XtremeGENE 9 transfection reagent (Roche Diagnostics) following manufacturer’s protocol. 24 h post-transfection, cells were treated for 3 h with cyclohexymide and actinomicyn D, fixed and mounted onto microscope slides using Vectashield mounting media containing DAPI (Vector). Using a Zeiss Axioskop fluorescence microscope, the subcellular localization of the fluorescent proteins was determined in at least 200 cells per sample. By applying the scoring system originally proposed^18^, the activity of functional NESs was rated between 1+ and 9+. The empty pRev(1.4)–GFP was used as a negative control in the assay.

On the other hand, the Quick-Change Lightning Site-Directed Mutagenesis Kit (Stratagene) was used to introduce the L144H mutation into a previously described mammalian expression plasmid encoding YFP-USP21^19^. HeLa cells were transfected with the plasmids encoding the wild-type and L144H mutant versions of YFP-USP21 NESs using XtremeGENE 9 transfection reagent. 24 h post transfection cells were fixed and processed for fluorescence microscopy as described above. Samples were analyzed using an Olympus Fluoview FV500 confocal microscope equipped with FV-viewer software, and the subcellular localization of YFP-USP21 (wt or mutant) was determined in 200 cells per sample.

## Additional Information

**How to cite this article**: Prieto, G. *et al*. Proteome-wide search for functional motifs altered in tumors: Prediction of nuclear export signals inactivated by cancer-related mutations. *Sci. Rep.* **6**, 25869; doi: 10.1038/srep25869 (2016).

## Supplementary Material

Supplementary Information

Supplementary Information

Supplementary Information

Supplementary Information

Supplementary Information

## Figures and Tables

**Table 1:** List of experimentally tested candidate NESs with mutation impact.

UniProt accession	Gene name	NES position	Wild type sequence	Mutant sequence	Wild type sequence score			Mutant sequence score (impact %)		
					Wregex	NESmapper	LocNES	Wregex	NESmapper	LocNES
O15523	(DDX3Y)	12.. 21	L-DQQ-L-AN-L-D-L	L-DQQ-L- **P** N-L-D-L	100	7.5	0.86	X (100)	0.0 (100)	0.77 (11)
O60437	(PPL)	967..976	L-QEE-L-EA-L-Q-L	L-QEE-L-EA- **Q** -Q-L	100	6.0	0.76	X (100)	X (100)	0.53 (31)
P08151	(GLI1)	495..503	L-RR-L-EN-L-R-L	L-RR- **R** -EN-L-R-L	100	5.5	0.74	X (100)	X (100)	X (100)
Q14C87	(TMEM132D)	668..678	L-GVQ-L-VTG-L-S-L	L-GVQ-L-VTG- **P** -S-L	86	7.0	0.79	X (100)	X (100)	0.42 (47)
Q9HAU5	(UPF2)	518..527	L-ELE-L-EN-L-E-I	L-ELE-L-EN-L-E- **S**	86	8.0	0.72	X (100)	X (100)	X (100)
P82094	(TMF1)	714..723	L-AIQ-V-GD-L-R-L	L-AIQ- **G** -GD-L-R-L	86	8.0	0.69	X (100)	X (100)	X (100)
Q9BZF9-2	(UACA)	529..538	L-KDQ-L-KD-L-K-V	**P** -KDQ-L-KD-L-K-V	74	7.5	0.74	X (100)	1.5 (80)	X (100)
O75899	(GABBR2)	805..814	L-DKD-L-EE-V-T-M	L-DKD-L-EE-V-T- **R**	74	6.0	0.70	X (100)	X (100)	0.31 (56)
Q9HCE9	(ANO8)	466..475	L-KEM-L-AT-L-L-I	L-KEM-L-AT-L- **P** -I	74	9.5	0.88	X (100)	X (100)	0.89 (−2)
Q14160	(SCRIB)	262..270	I-GQ-L-KQ-L-S-I	I-GQ- **R** -KQ-L-S-I	74	6.0	0.88	X (100)	1.0 (83)	X (100)
O75449	(KATNA1)	391..400	L-RIS-L-RE-L-E-L	L-RIS-L-RE- **W** -E-L	100	6.5	0.69	X (100)	0.5 (92)	X (100)
Q9UK80	(USP21)	135..144	L-GAA-L-SR-L-A-L	L-GAA-L-SR-L-A- **H**	100	3	0.53	X (100)	X (100)	0.31 (41)

All candidates are within the first quartile in the three NES predicting tools and have a mutation impact greater than 80% in at least two of the tools.
