# Supplementary material for: Proteome-wide search for functional motifs altered in tumors: Prediction of nuclear export signals inactivated by cancer-related mutations
Source: Sci Rep. 2016 May 12;6:25869. doi: 10.1038/srep25869 (PMC4865848; doi:10.1038/srep25869)
Supplement: Supplementary Information [file srep25869-s1.pdf]

Supplementary Material

Proteome-wide search for functional motifs altered in tumors. Prediction of nuclear export signals inactivated by cancer-related mutations

Gorka Prieto, Asier Fullaondo, Jose A. Rodríguez

December 3, 2015

Supplementary figures

1 Mutation impact of predicted monopartite NLS motifs for the whole human proteome 2

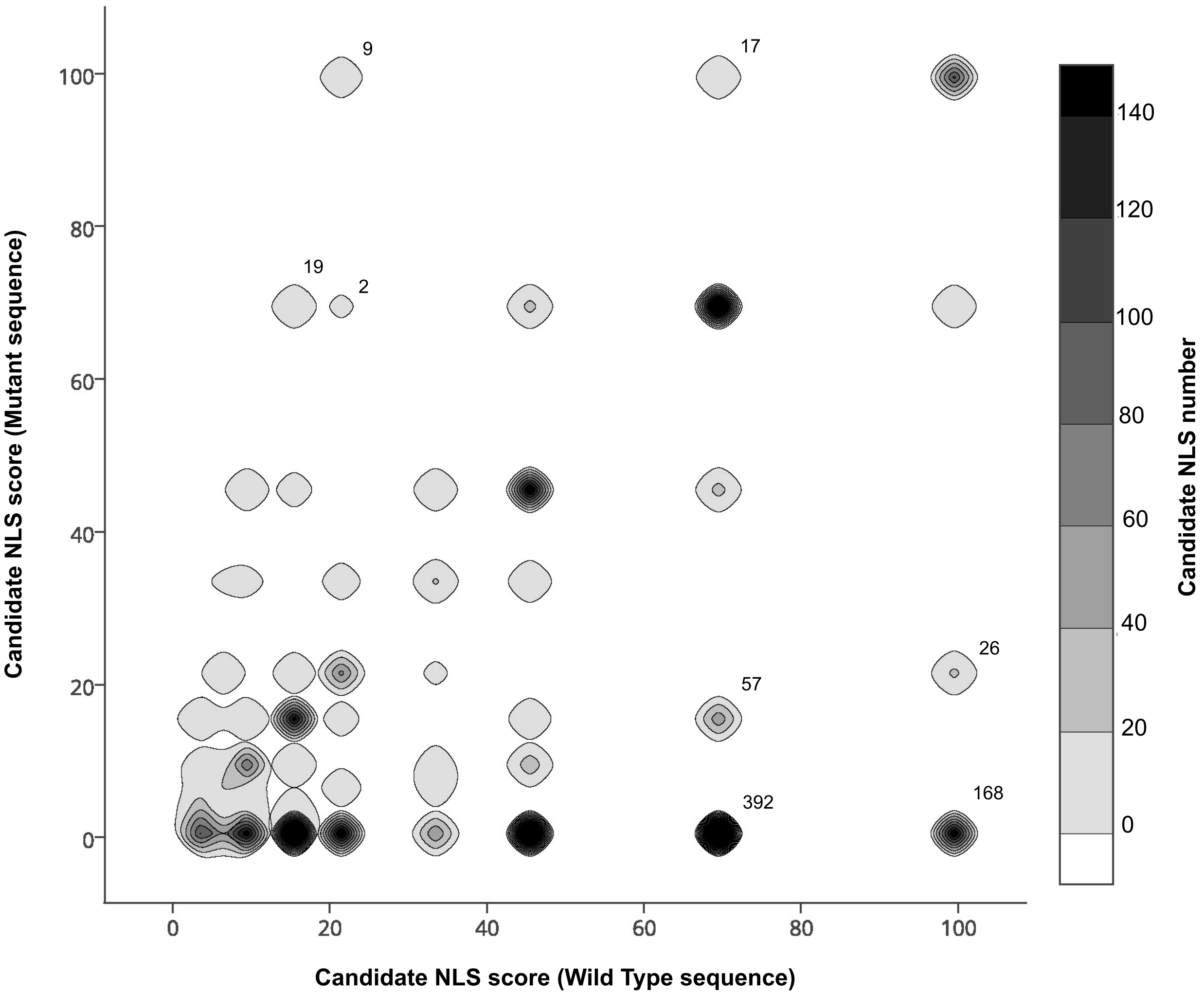

Supplementary figure 1: Mutation impact of predicted monopartite NLS motifs for the whole human proteome. Wregex was used to search the whole human proteome from UniProt/SwissProt (release 2015_04), using the NLS_MonoCore motif, and enabling the COSMIC (release v71) mutations support. This NLS_MonoCore motif is based on the regular expression available at ELM (accession ELME000270) and a PSSM trained with the 17 instances available there. This search resulted into 3172 COSMIC missense mutations altering potential NLS motifs. In the x-axis the Wregex score of the wild type candidate sequence is displayed. The y-axis displays the Wregex score of the candidate sequence after substituting an amino acid with a COSMIC missense mutation. Points near the diagonal line indicate no significative difference between the mutant and wild type sequences predicted scores. Points above the line indicate mutants with NLS activity gain, and the points below are mutants with NLS activity loss. We can see that the candidates distribution is different than the one for NES depicted in Figure 3. Now more mutations have a significative impact in the predicted score because the monopartite NLS motif definition is more strict than the one used for NES.
